# Hidden in the Rash: A Sneddon Syndrome Case Report

**DOI:** 10.7759/cureus.50562

**Published:** 2023-12-15

**Authors:** Rema A Almutawa, Hassan M Almalak, Mohammed E Alotaibi

**Affiliations:** 1 College of Medicine, King Saud University, Riyadh, SAU; 2 Department of Internal Medicine, King Saud University Medical City, Riyadh, SAU; 3 Department of Medicine, Faculty of Medicine, College of Medicine, King Saud University Medical City, Riyadh, SAU

**Keywords:** paroxysmal hemicrania, stroke, cerebro-vascular accident, livedo racemoasa, sneddon syndrome

## Abstract

This clinical case report aims to highlight the unusual presentation of Sneddon syndrome with a possible association with paroxysmal hemicrania. A medical record review was performed at a tertiary hospital in Riyadh, Saudi Arabia. Data collected include clinical evaluations and laboratory and imaging results. Informed consent was obtained. Hereby, we present a 27-year-old female who presented with multiple stroke attacks, along with severe headaches involving right retro-orbital pain with an eight-year history of spotted skin lesions. Initial unenhanced computed tomography (UCT) brain in the emergency showed left insular cortex hypodensity, revealing acute ischemic insult. Subsequent magnetic resonance imaging (MRI) and magnetic resonance angiography (MRA) revealed acute ischemic infarct in the territory of the left middle cerebral artery (MCA) involving the insula and frontoparietal lobe. Further investigations were done, including cerebrospinal fluid (CSF) analysis and autoimmune and infectious workup, which were unrevealing. Skin biopsy of the lesions showed subcutaneous fat necrosis with nonspecific scattered fibrinogen positivity and was labeled as livedo reticularis vs. livedo racemosa. A Sneddon syndrome diagnosis can be very challenging, needing a high index of suspicion to direct the diagnostic investigations. Moreover, the presence of a severe headache is an unusual phenomenon that needs further study.

## Introduction

Sneddon syndrome is a rare, non-inflammatory, occlusive vasculopathy affecting small- and medium-sized arteries of the brain and skin [1,2]. Sneddon syndrome can present with stroke, transient ischemic attack, livedo reticularis, and progressive dementia [1,2]. Very few cases reported a headache as one of the symptoms with no clear association. Hereby, we present an unusual case of Sneddon syndrome with the possible association with paroxysmal hemicrania.

## Case presentation

We report a case of a 27-year-old Saudi female, a known case of primary Raynaud’s disease for more than 10 years on a nitroglycerin patch. She was in her usual state of health till a week prior to her presentation when she started to have on-and-off episodes of vertigo, along with right upper limb numbness. She presented to our emergency department with a one-day history of dysarthria, facial asymmetry, and right upper limb weakness. She denied having visual symptoms, unsteady gait, abnormal movement, or loss of consciousness. She also complained of a holocephalic headache for a long time, throbbing in nature, lasting for hours, and then resolved, which is not associated with nausea and vomiting, photophobia, or phonophobia and does not affect her daily activity. A review of systems revealed having right index finger swelling and joint pain.

During her first presentation, she was vitally stable. During examination, she was alert, attentive, and oriented to time, place, and person. She showed impaired naming and reading, but intact comprehension, repetition, and writing. Her neurological examination revealed reduced pinprick sensation over the right trigeminal branches (V1, V2, and V3), absent gag reflex, right facial droop sparing the forehead, reduced power and sensations in the right upper limb (RUL) with spastic tone, positive Hoffman's sign in RUL, normal reflexes all over, but brisker in RUL, and normal gait and coordination. Her pupils were equally reactive and non-dilated, measuring 3 mm bilaterally. Other system examinations revealed redness over her cheeks and nose and cold feet with blue discoloration over the right big toe.

Her laboratory investigations were unremarkable. Unenhanced computed tomography (CT) brain showed left insular cortex hypodensity, concerning for an acute ischemic insult, with no intracranial hemorrhage. Therefore, the patient was started on aspirin 81 mg. Her working diagnosis was a stroke at a young age. CT arterial and venous angiogram was done and showed patent major intracranial arteries, with no flow-limiting stenosis and no evidence of cerebral venous thrombosis.

An extensive workup was done. A lumbar puncture was performed. The sampled cerebrospinal fluid (CSF) looked clear, with normal cells. The echocardiogram and Holter monitor were both normal. An autoimmune workup was done, which was unrevealing (antinuclear antibodies, antinuclear cytoplasmic antibodies, anti-proteinase 3 antineutrophil cytoplasmic antibody, myeloperoxidase, complement levels, inflammatory markers). A thrombophilia workup was unremarkable, except for protein S, which was 39% (low normal: 55%). An infectious workup was unremarkable.

**Figure 1:**
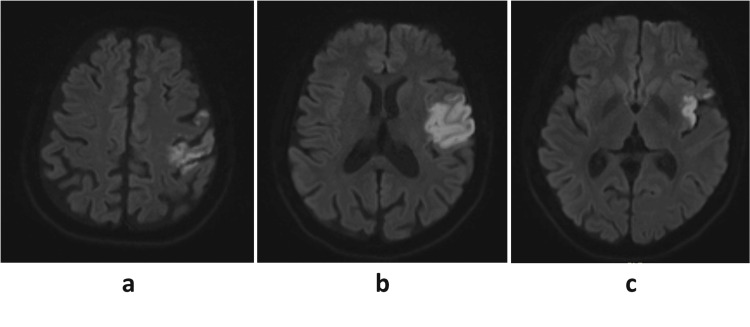
Axial magnetic resonance imaging (DWI) Axial magnetic resonance shows restricted diffusion along the left middle cerebral artery territory involving the (a & c) insula and (b) frontoparietal lobe.

**Figure 2:**
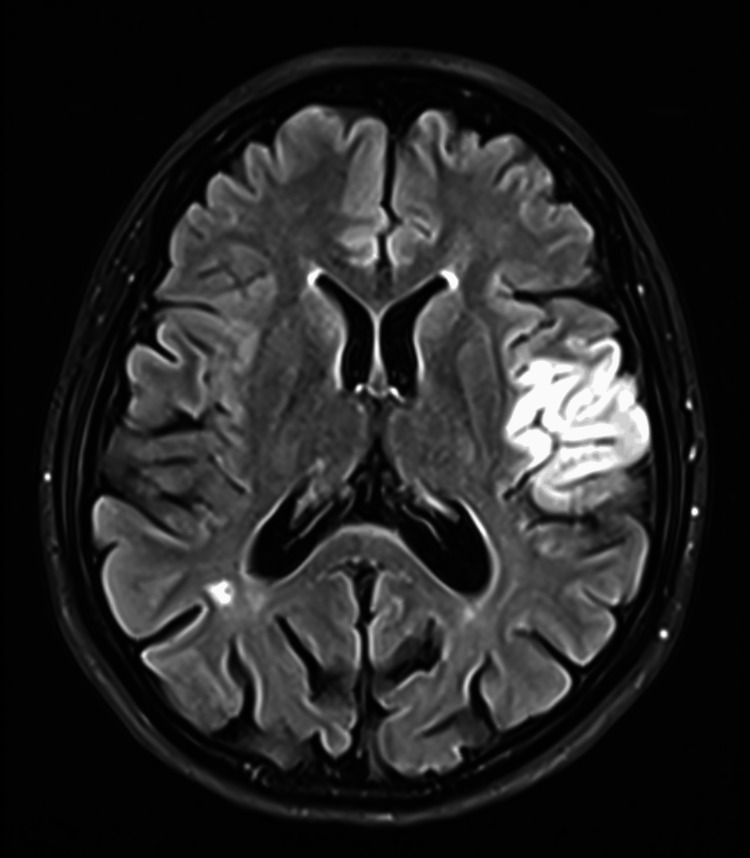
Axial magnetic resonance imaging (Flaire) Axial magnetic resonance (Flaire) shows the acute ischemic infarct in the territory of the left middle cerebral artery (MCA) involving the insula and frontoparietal lobe extending to the pre- and post-central gyri.

**Figure 3:**
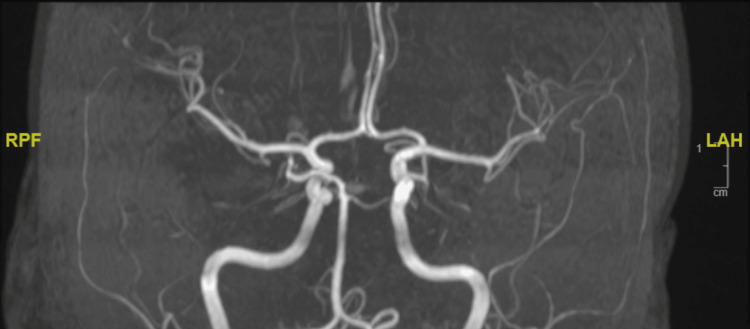
Magnetic resonance angiography (MRA) Magnetic resonance angiography (MRA) shows the patent bilateral middle cerebral arteries with no limitation of flow or stenosis.

Magnetic resonance imaging (MRI) (Figures 1-2) and magnetic resonance angiography (MRA) (Figure 3) of the brain showed acute ischemic infarct in the territory of the left middle cerebral artery (MCA) involving the insula, and the frontoparietal lobe extends to the pre- and postcentral gyri. There is no acute intracranial hemorrhage or petechial hemorrhage, but features of chronic small vessel disease. Interventional radiology was unremarkable for any occlusion.

MRI and MRA Tesla-3 were done and showed the expected evolution of the left MCA subacute infarction. Multiple old lacunar infarcts in the cerebral white matter and deep gray matter with associated bilateral cerebral atrophy, predominantly involving the high frontal and parietal convexities. There was callosal involvement. Features of retinal and vestibulocochlear involvement were appreciated.

During her stay, the patient showed improvement in her weakness and dysarthria. Upon discharge, the patient resolved completely, except for slight facial deviation, with decreased sensation over the trigeminal branches and residual right-sided weakness. The patient was referred to occupational and physical therapy for assessment. The patient was started on amitriptyline.

She presented to the emergency department multiple times with the same three layers of deterioration. Her headache was associated with right eye pain phonophobia and right-sided numbness involving her upper and lower limbs. The patient was started on dual antiplatelet therapy (DAPT) for 21 days and then to complete aspirin for life. CT/CTA was unremarkable for acute insult with no large vessel occlusion on CTA. MRI brain showed no new ischemic insult, with no diffusion restriction. The patient was also evaluated by the dermatology team because of spotted skin for almost eight years along the extremities and was labeled as livedo reticularis vs. livedo racemosa. A skin biopsy was performed and showed subcutaneous fat necrosis with nonspecific scattered fibrinogen positivity. The query of Sneddon syndrome +/- hemicrania paroxysmal was raised.

Following up, the patient reported no recurrence of symptoms. She had mild spasticity over the right upper limb with improved power, as shown in Table 1.

**Table 1:** Power of the right upper limb Medical Research Council (MRC) scale for muscle strength (0-5): 5: Normal 4: Movement against gravity and resistance 3: Movement against gravity over the full range 2: Movement of the limb but not against gravity 1: Visible contraction without movement 0: No visible contraction

Power of the right upper limb (MRC scale for muscle strength)			
	First presentation	Upon discharge	Follow-up
Finger extension	3/5	5/5	5/5
Finger flexion	3/5	5/5	5/5
Finger abduction	3/5	3/5	4/5
Wrist extension	4/5	5/5	4/4
Wrist flexion	4-/5	5/5	4/5
Elbow extension	5/5	5/5	5/5
Elbow flexion	4/5	5/5	5/5
Shoulder abduction	4/5	+4/5	5/5
Shoulder adduction	5/5	5/5	5/5

## Discussion

Sneddon syndrome is a non-inflammatory vasculopathy involving small- and medium-sized arteries. Sneddon syndrome involves various clinical features, including neurological and non-neurological symptoms [3,4]. The disease commonly affects women aged 20-40 years. Multiple isolated familial cases were reported suggesting genetic causes [4].

The most important neurological manifestations are recurrent ischemic stroke manifestations, such as hemiparesis, sensory disturbances, aphasia, and visual field defects [3]. The most common involved territory is the superficial MCA [3-5]. MRI usually demonstrates small and multifocal lesions in the white matter mostly located in the periventricular area. MRI findings are nonspecific for Sneddon syndrome and must be interpreted in correlation with clinical manifestations [6]. Cerebral angiograms usually show stenosis and/or occlusion of the small- and medium-sized arteries and may show leptomeningeal and transdural collateral networks [3]. Other reported neurological symptoms include diffuse cortical atrophy, early-onset dementia, psychiatric disturbances, migraines, epilepsy, and intracranial hemorrhage [3,4]. Cognitive dysfunction was shown to occur in almost 77% of patients with Sneddon syndrome [5]. Headache is the most reported symptom in almost 50% of patients [3,6]. Two cases were reported describing the headaches experienced by these patients [1,2]. In our observation, headache attacks seem to be holocephalic and with no reported symptoms of (nausea, vomiting, photophobia, and phonophobia). Our patient showed new features of the headache in the subsequent Sneddon attacks involving severe retro-orbital pain associated with throbbing headache directing the diagnosis into paroxysmal hemicrania. Martinelli et al. concluded that headache attacks can be the first isolated neurological symptoms of the syndrome before developing other symptoms [1]. Headaches are described as dull and diffuse and usually precede the neurological symptoms for two months to 15 years [6]. The most common non-neurological manifestation is livedo racemosa, which is an erythematous netlike broken irregular rash that is persistent on rewarming due to occlusion of small- and medium-sized arteries in comparison to livedo reticularis, which has a continuous netlike pattern that resolves with rewarming [3,5]. Livedo racemosa usually appears many years before the onset of stroke [3]. Other dermatological manifestations include Raynaud’s phenomena and widespread cutaneous discoloration because of systemic angiomatosis [3,7].

Laboratory findings in Sneddon syndrome patients are similar to patients with antiphospholipid syndrome. Elevated antiphospholipid antibody (aPL) levels are found in almost 57% of patients with Sneddon syndrome [7]. Thrombocytopenia is commonly found in patients with positive antiphospholipid antibodies [7]. Skin biopsy can be used to help direct the diagnosis of Sneddon syndrome. Histopathological findings include endotheliitis, fibrin thrombi occluding vessels, and intima and media proliferation without evidence of vasculitis involving mostly arteries of the reticular dermis [4]. The diagnosis of Sneddon syndrome requires the combination of neurological, dermatological, and neuroradiological findings, which all together may raise the suspension of a Sneddon syndrome diagnosis [6].

In aPL-positive patients, no significant difference was found between anticoagulants and antiplatelets to decrease the risk of stroke recurrence, although anticoagulants seem to be more effective [3]. In aPL-negative patients, no difference was found between anticoagulants and antiplatelets, so the choice of antithrombotic treatment should be individualized [5]. In our case, our patient was compliant on aspirin with good follow-up and no stroke recurrence.

## Conclusions

In conclusion, Sneddon syndrome is a small vessel vasculitis with neurological and non-neurological symptoms. Only a few reports reviewed the relationship between Sneddon syndrome and headaches. The nature of the disease is still not completely understood. Unfortunately, there is no cure or specific treatment.

## References

[1] Martinelli A, Martinelli P, Ippoliti M, Giuliani S, Coccagna G (1991). Sneddon syndrome presenting with hemicranic attacks: a case report. Acta Neurol Scand.

[2] Cavestro C, Richetta L, Pedemonte E, Asteggiano G (2009). Sneddon's syndrome presenting with severe disabling bilateral headache. J Headache Pain.

[3] Samanta D, Cobb S (2020). Freiberg’s infarction as the first clinical presentation of Sneddon syndrome. J Pediatr Neurosci.

[4] Jiménez-Gallo D, Albarrán-Planelles C, Linares-Barrios M, González-Fernández JA, Espinosa-Rosso R, Báez-Perea JM (2014). Sneddon syndrome presenting with unilateral third cranial nerve palsy. J Neuroophthalmol.

[5] Cleaver J, Teo M, Renowden S, Miller K, Gunawardena H, Clatworthy P (2019). Sneddon syndrome: a case report exploring the current challenges faced with diagnosis and management. Case Rep Neurol.

[6] Stockhammer G, Felber SR, Zelger B, Sepp N, Birbamer GG, Fritsch PO, Aichner FT (1993). Sneddon's syndrome: diagnosis by skin biopsy and MRI in 17 patients. Stroke.

[7] Marinho JL, Piovesan EJ, Leite Neto MP (2007). Clinical, neurovascular and neuropathological features in Sneddon's syndrome. Arq Neuropsiquiatr.

